# Magnetic Seizure Therapy for Suicidality in Treatment-Resistant Depression

**DOI:** 10.1001/jamanetworkopen.2020.7434

**Published:** 2020-08-18

**Authors:** Cory R. Weissman, Daniel M. Blumberger, Julia Dimitrova, Alanah Throop, Daphne Voineskos, Jonathan Downar, Benoit H. Mulsant, Tarek K. Rajji, Paul B. Fitzgerald, Zafiris J. Daskalakis

**Affiliations:** 1 Temerty Centre for Therapeutic Brain Intervention, Centre for Addiction and Mental Health, Department of Psychiatry, University of Toronto, Toronto, Ontario, Canada; 2 Krembil Research Institute, Toronto Western Hospital, University Health Network, Toronto, Ontario, Canada; 3 Department of Psychiatry, University of Toronto, Toronto, Ontario, Canada; 4 Centre for Addiction and Mental Health, Department of Psychiatry, University of Toronto, Toronto, Ontario, Canada; 5 Epworth Centre for Innovation in Mental Health, Epworth Healthcare and Monash University Department of Psychiatry, Camberwell, Victoria, Australia

## Abstract

**Importance:**

There is an unmet need for effective treatments for suicidality in mental disorders. Magnetic seizure therapy (MST) has been investigated as an alternative to electroconvulsive therapy, a known effective treatment for suicidality, in the management of treatment-resistant major depressive disorder, with promising findings. Yet, there are very limited data on the association of MST with suicidality directly. It is important to explore the potential of MST as a viable treatment alternative to electroconvulsive therapy for suicidality.

**Objective:**

To determine the association of MST with suicidality in patients with treatment-resistant major depressive disorder.

**Design, Setting, and Participants:**

This nonrandomized controlled trial took place at a single tertiary care psychiatric facility in Canada. It followed an open-label study design with consecutive treatment cohorts. Consecutive groupings of 67 patients with treatment-resistant major depressive disorder and with baseline suicidality present were treated for up to 24 treatments. The study was run from February 2012 through June 2019. Patients were followed up for 6 months at the end of the treatment period. This post hoc secondary analysis of the trial was performed from January to November 2019.

**Interventions:**

MST was delivered at 100% stimulator output over the prefrontal cortex with low (25 Hz), moderate (50 or 60 Hz), or high (100 Hz) frequency, for a maximum of 24 sessions.

**Main Outcomes and Measures:**

Remission from suicidality was measured as an end point score of 0 on the Beck Scale for Suicidal Ideation. A linear mixed model was used to assess the trajectory of Beck Scale for Suicidal Ideation scores.

**Results:**

A total of 67 patients (mean [SD] age, 46.3 [13.6] years; 40 women [60.0%]) received a mean (SD) of 19.5 (5.1) MST treatments. The overall number of patients achieving remission was 32 (47.8%). Sixteen patients (55.2%) receiving low-frequency MST achieved remission, as well as 12 patients (54.5%) in the moderate-frequency group, and 4 patients (25.0%) in the high-frequency group. The linear mixed model revealed an association of time with Beck Scale for Suicidal Ideation scores (*F*_8,293.95_ = 5.73; *P* < .001).

**Conclusions and Relevance:**

These findings suggest that MST may be an effective treatment for suicidality, and sensitivity analysis shows this may be particularly so at low and moderate frequencies. Future studies should directly compare MST with electroconvulsive therapy for treating suicidality and should evaluate MST as a treatment for suicidality across mental disorders.

**Trial Registration:**

ClinicalTrials.gov Identifier: NCT01596608

## Key Points

**Question:**

Is magnetic seizure therapy associated with decreased suicidality in patients with treatment-resistant depression?

**Findings:**

This nonrandomized controlled trial of 67 patients in consecutive cohorts treated with magnetic seizure therapy found an overall remission rate from suicidality of 47.8%. Remission rates were higher in the low- and moderate-frequency treatment groups compared with the high-frequency group.

**Meaning:**

These findings suggest that magnetic seizure therapy holds early promise as a treatment for suicidality in patients with treatment-resistant depression.

## Teaser

This nonrandomized controlled trial examines the association of magnetic seizure therapy with suicidality in patients with treatment-resistant depression.

## Introduction

Suicidality, a term that encompasses the spectrum of suicidal thoughts and behaviors, is a major public health problem. Worldwide, at least 800 000 people die by suicide each year.^1^ Approximately 90% of these individuals who die by suicide have a primary psychiatric illness.^2^ For patients with unipolar and bipolar depression, the lifetime rate of suicide is 15% to 20%.^3^ As such, there is a considerable need for new, effective, and better-tolerated treatments for suicidality in patients with both subacute and emergent suicidality.

Evidence-based pharmacological treatments for both subacute and emergent suicidality are limited. Clozapine has known antisuicidal effects but is indicated only for patients with treatment-resistant schizophrenia.^4^ Lithium has robust protective effects against suicide, but its use is typically limited to patients with bipolar disorder.^5^ Ketamine is a promising antisuicidal treatment,^6^ but it appears to have transient effects and is still experimental. In addition, ketamine may have addiction potential,^6,7^ possibly through intrinsic opioid agonism activity.^8^ Commonly used antidepressant medications are not consistently protective against suicide^9^ and may even increase suicidality in youths.^10^ Electroconvulsive therapy (ECT) is a very effective treatment for suicidality in mood disorders, with various forms of evidence supporting this claim dating back more than 80 years.^11,12,13^ In the landmark Consortium for Research in ECT Study,^11^ ECT led to rapid remission of high expressed suicidality in 250 patients with major depressive disorder and bipolar depression, with a suicidality remission rate of 63.2%. Although ECT is highly effective at treating suicidality, it is an underused treatment: fewer than 1% of patients with treatment-resistant depression (TRD) receive ECT.^14^ This is because of a combination of stigma and perceived risk of cognitive adverse effects.^15,16^

Effective and tolerable treatments for suicidality are needed. New lines of research are being created to explore alternative forms of brain stimulation for treating suicidality. Recent evidence suggests a potential role for repetitive transcranial magnetic stimulation (rTMS)^17,18,19^ and transcranial direct current stimulation^20^ as treatments for subacute suicidal ideation through targeting of the brain region the dorsolateral prefrontal cortex. Magnetic seizure therapy (MST) is another emerging brain stimulation therapy in which magnetic pulses, similar to rTMS, induce focal seizures, similar to ECT, in patients under general anesthesia.^21^ The hope for MST is to match the treatment efficacy of ECT with fewer adverse effects, because its effect is mediated by a different mechanism of action and a more focal treatment target in the brain structures.^21,22^ There is evidence for this with regard to MST as a treatment for major depressive disorder.^22,23,24^ However, it has not yet been established whether MST has the same antisuicidal effects as ECT. Thus, we explored the association of MST with suicidality in a secondary analysis of a recently published open-label study on MST for TRD.^22^ We hypothesized that MST would be associated with clinically meaningful rates of remission from suicidality as measured by the Beck Scale for Suicidal Ideation (SSI). We also explored whether the association of MST with suicidality differed among different MST stimulation frequencies in a sensitivity analysis.

## Methods

### Overall Design

This study is a secondary analysis of data from an open-label, nonrandomized, controlled trial of MST as a treatment for TRD, which is described in detail in the original report^22^ (see the eAppendix in the Supplement for the full clinical trial protocol). This study follows the Transparent Reporting of Evaluations With Nonrandomized Designs (TREND) guideline for reporting of nonrandomized evaluations.^25^

### Participants

As described previously,^22^ all participants were aged 18 to 85 years, presented with TRD, and were initially referred for a course of ECT. The research protocol was approved by the Centre for Addiction and Mental Health Research ethics board in accordance with the Declaration of Helsinki, and all patients provided written informed consent.^22^ The study was run from February 2012 through June 2019. All patients had a baseline score on the 24-item Hamilton Rating Scale for Depression (HRSD-24) of 21 or higher. Women of child-bearing potential had to be using an accepted form of contraception. Exclusion criteria were as follows: unstable physical or neurological illness or other significant neuropsychiatric comorbidity; currently pregnant or lactating; not stable enough to undergo general anesthesia; having a cardiac pacemaker, cochlear implant, implanted electronic device, or nonelectric metallic implant; use of any anticonvulsant or a benzodiazepine at a dosage equivalent to lorazepam 2 mg per day or higher; active substance misuse during the preceding 3 months; a diagnosis of delirium, dementia, or cognitive disorder secondary to general medical condition; and history of an eating disorder, borderline personality disorder, or antisocial personality disorder. Patients with a suicide attempt during the previous 6 months were also excluded from the study.

**Figure 1.**
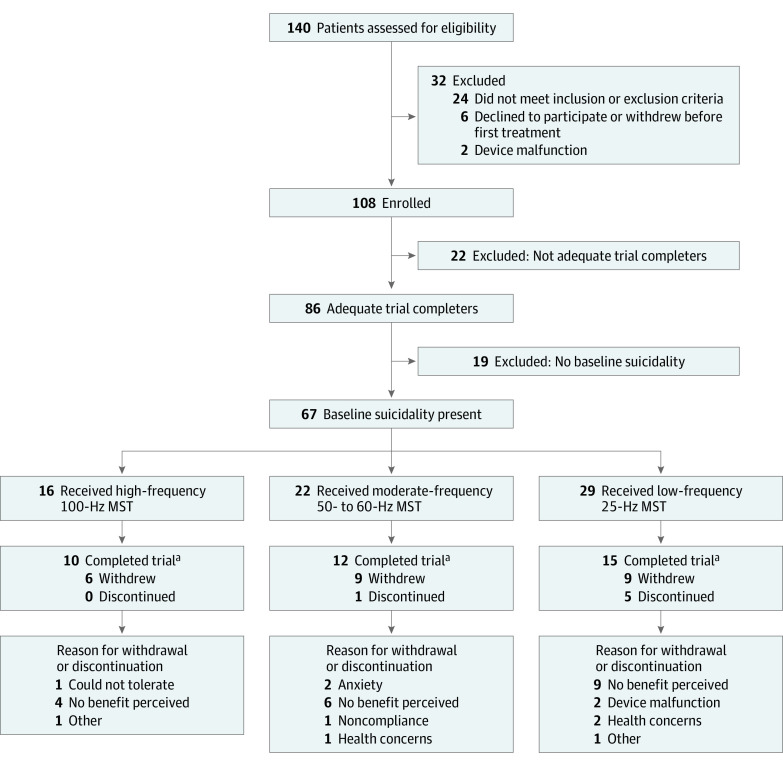
Patient Flow Diagram Adapted from Daskalakis et al,^22^ 2020. MST indicates magnetic seizure therapy. ^a^Patients completed trial as per primary depression outcome.

In the original study,^22^ a total of 86 participants with a *Diagnostic and Statistical Manual of Mental Disorders* (Fourth Edition, Text Revision) major depressive episode with or without psychotic features completed an adequate MST trial. Nineteen of these 86 participants (22.1%) did not have baseline suicidality, as defined by a score greater than 0 at the initial study visit on the Beck SSI, a validated 19-item scale used to quantify suicidality with a range from 0 to 38.^26^ The main analyses in the present report focus on the 67 participants with baseline suicidality who were defined as adequate trial completers in that they completed at least 8 MST sessions. In addition, we analyzed outcomes in a subgroup of 36 participants defined as protocol completers: either they attained remission from depression or they completed the maximum number of MST sessions selected a priori, 24. Please refer to Figure 1 for the modified CONSORT diagram.

### MST Procedures

Participants completed MST sessions 2 to 3 times per week until they achieved remission from their depressive episode or until they reached a maximum of 24 sessions. Participants received MST using the MagPro MST device with Twin Coil-XS (both from MagVenture) applied over the frontal cortex at 100% machine output with low (25 Hz), moderate (50 or 60 Hz), or high (100 Hz) frequency. These frequencies were chosen for participants in an open-label fashion in consecutive treatment cohorts (ie, there was no randomization for treatment groupings). Please refer to the original report for further details of treatment procedures.^22^

### Outcome Measures

Participants were assessed every 3 weeks and at the end of the study with the Beck SSI and HRSD-24. Participants were also followed for 6 months at the end of the treatment period. The primary outcome for this study was remission from suicidality as measured by an end point score of 0 on the Beck SSI.^26^ To cross-validate our results with the Beck SSI, we also report outcomes with the suicide item (item 3) of the HRSD-24, which has also been used in previous studies of suicidality.^27^

### Statistical Analysis

Statistical analysis was completed using SPSS statistical software version 24 (IBM Corp). All analyses were 2-tailed with the significance level set as *P* < .05 for all outcomes. Post hoc *t* tests were completed when significant results were found with analysis of variance or χ^2^ analyses. We calculated Pearson correlation coefficients for change in the HRSD-24 suicide item and change in the Beck SSI score. Linear mixed models were used with Beck SSI scores as dependent continuous measures. These analyses were conducted using the MIXED command in SPSS. The basic model included treatment frequency group, time, and the interaction between group and time. Model fit was compared with and without the following covariates, reflecting the degree of treatment resistance: mean cumulative Antidepressant Treatment History Form (ATHF) score (derived by adding the ATHF ratings of all antidepressant trials during the current episode), number of adequate antidepressant trials in the current episode (ie, trials rated ≥3 on the ATHF), and the total number of psychotropic medication trials. As a subgroup type sensitivity analysis,^28,29^ we analyzed differences between groupings by treatment frequency. We compared baseline characteristics between these groups and calculated effect size (Cohen *d*) for the primary outcome for each treatment frequency grouping. Data analysis was performed from January to November 2019.

## Results

### Patient Flow, Demographic Characteristics, and Follow-up

A total of 67 patients (mean [SD] age 46.3, [13.6] years; 40 women [60.0%]) received an mean (SD) of 19.5 (5.1) MST treatments. Table 1 presents the demographic and clinical characteristics of the 67 adequate trial completers divided into treatment frequency groups. Of the 67 participants who had baseline suicidality present and completed a minimally adequate MST trial, 36 (53.7%) completed the protocol. None of the participants attempted or completed suicide during the study or the 6-month follow-up period. One participant with no baseline suicidality experienced the emergence of suicidality during the course of MST, with a change in Beck SSI score from 0 to 4 of a maximum possible score of 38. The 3 treatment frequency groups did not differ in terms of baseline suicidality (Beck SSI scores) (*F*_2,64_ = 0.68; *P* = .52) or in other variables, except for their baseline cumulative ATHF score (*F*_3,72_ = 3.53; *P* = .02): the scores of the high-frequency group were significantly lower than the scores of both the low-frequency group (*t*_40_ = 2.02; *P* < .001) and the moderate-frequency group (*t*_24_ = 2.06; *P* = .003).

**Table 1.** Demographic and Clinical Variables by Treatment Frequency in Adequate Trial Completers Group^a^

Variable	Mean (SD)^b^			
	Total (N = 67)	Treatment frequency		
		Low (n = 29)	Moderate (n = 22)	High (n = 16)
Female, No. (%)	40 (60.0)	15 (52.0)	15 (68.0)	10 (63.0)
Education, y	15 (3.10)	15.28 (2.75)	14.81 (3.08)	16 (3.79)
Age, y	46.3 (13.6)	46.2 (13.1)	47.2 (14.0)	45.4 (14.8)
Age at onset of major depressive disorder, y	23.7 (11.6)	23.8 (12.1)	25.6 (13.5)	20.8 (7.5)
Length of current major depressive episode, wk	175.3 (185.6)	178.7 (193.5)	203.4 (186.2)	130.6 (172.8)
Recurrent major depressive episodes, No. (%)	52 (78.0)	23 (79.0)	15 (68.0)	14 (88.0)
Antidepressant treatment history form cumulative score	13.1 (9.1)	15.3 (10.3)^c^	14.5 (8.3)^c^	7.1 (4.0)^c^
Magnetic seizure therapy sessions, No.	19.5 (5.1)	19.5 (5.2)	19.8 (4.8)	19.31 (5.4)

^a^ Adequate trial completers are defined as participants completing 8 or more magnetic seizure therapy sessions.

^b^ Magnetic seizure therapy frequencies are defined as low (25 Hz), moderate (50 or 60 Hz), and high (100 Hz).

^c^ Denotes statistically significant differences (*P* < .05).

### Adequate Trial Completers

The overall number of patients achieving remission from suicidality was 32 of 67 (47.8%) among the adequate trial completers. The mean (SD) Beck SSI score decreased from 10.9 (4.9) at baseline to 6.0 (6.6) at the end of the trial (paired *t*_66_ = 2.0; *P* < .001). Analysis with the suicide item of the HRSD-24 validated these results with qualitatively similar outcomes (eTable in the Supplement). The change in Beck SSI scores and HRSD-24 suicide item scores were moderately correlated for the 67 patients (*r* = 0.47; *P* < .001).

**Figure 2.**
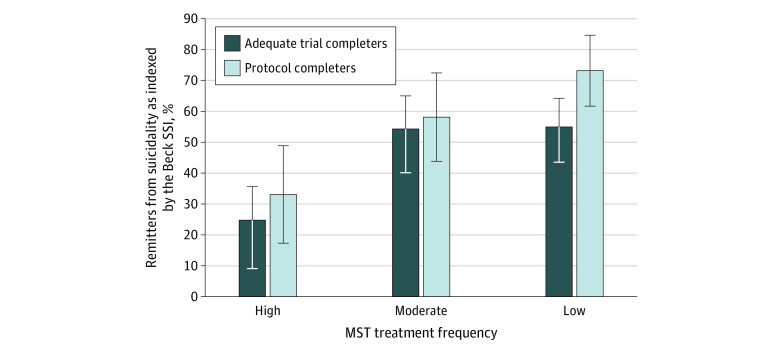
Rates of Remission From Suicidality Indexed by Beck Scale for Suicidal Ideation (SSI) Scores in the Adequate Trial Completers and Protocol Completer Groups There were 67 patients in adequate trial completer group (ie, participants who completed ≥8 magnetic seizure therapy [MST] sessions) and 36 patients in the protocol completer group (ie, those who attained remission from depression or completed 24 MST sessions, which is the maximum). MST frequencies are defined as low (25 Hz), moderate (50 or 60 Hz), and high (100 Hz). Vertical lines and error bars indicate SEs.

For the subgroup sensitivity analysis, participants treated with low-frequency and moderate-frequency MST had numerically higher rates of remission from suicidality (16 participants [55.2%] and 12 participants [54.5%], respectively) than those treated with high-frequency MST (4 participants [25.0%]) (Figure 2); however, the differences between these rates did not reach statistical significance (low vs high frequency, χ^2^_1_ = 3.802; *P* = .05). The effect size for change in Beck SSI scores were large and statistically significant in the 2 groups treated with low-frequency (Cohen *d* = 1.43; 95% CI, 0.59 to 1.88) and moderate-frequency (Cohen *d* = 0.87; 95% CI, 0.28 to 1.35) MST. In the high-frequency MST treatment group, the effect size was not significant (Cohen *d* = 0.42; 95% CI, −0.35 to 1.04) (Table 2).

**Table 2.** Suicidality Scores by Treatment Frequency for Adequate Trial Completers^a^

Variable	Treatment frequency^b^		
	Low (n = 29)	Moderate (n = 22)	High (n = 16)
Beck Scale for Suicidal Ideation score, mean (SD)			
At baseline	10.3 (5.5)	11.0 (4.8)	12.1 (3.9)
At end point	4.7 (6.2)	5.0 (6.8)	9.0 (6.5)
Relative reduction, %	53.4	64.4	18.8
Rate of remission from suicidality %^c^	55.2	54.5	25.0

^a^ Adequate trial completers are defined as participants completing 8 or more magnetic seizure therapy sessions.

^b^ Magnetic seizure therapy frequencies are defined as low (25 Hz), moderate (50 or 60 Hz), and high (100 Hz).

^c^ Remission from suicidality is defined as a final score of 0.

**Figure 3.**
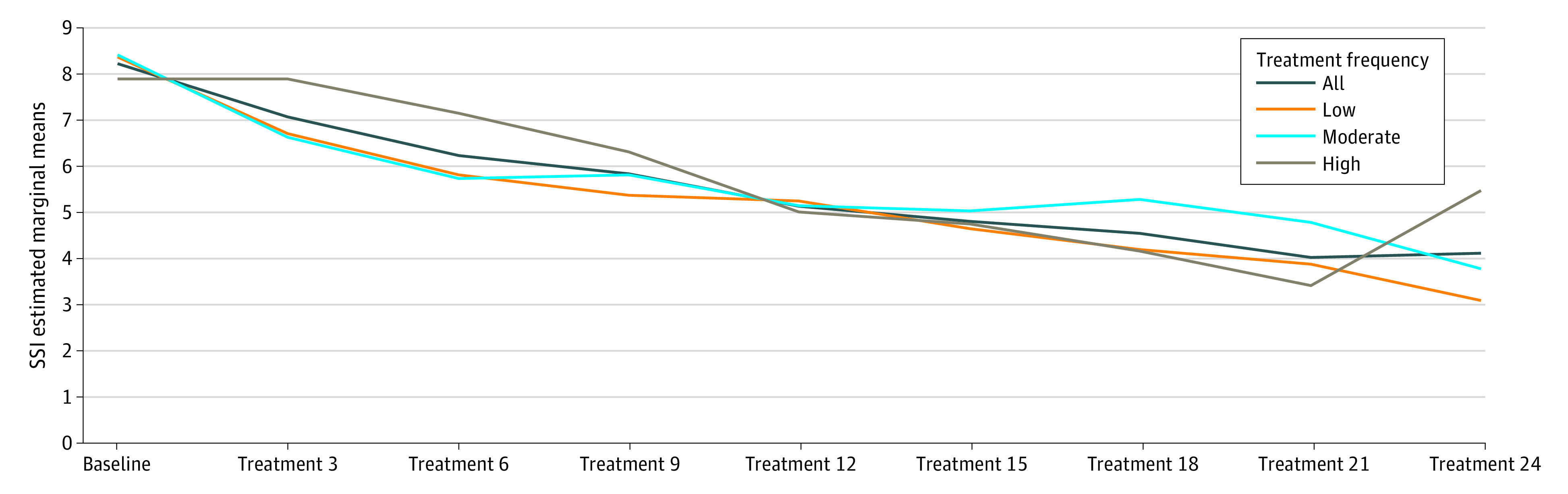
General Linear Mixed Model of Beck Scale for Suicidal Ideation (SSI) Total Scores by Treatment Frequency With Group-by-Time Interaction Magnetic seizure therapy frequencies are defined as low (25 Hz), moderate (50 or 60 Hz), and high (100 Hz).

The linear mixed model revealed a main association of time (*F*_8,293.95_ = 5.73; *P* < .001) with SSI scores. In the sensitivity analysis, the group-by-time interaction was not statistically significant (Figure 3). The goodness-of-fit of the model improved when we included the following covariates: mean cumulative ATHF score, number of adequate antidepressant trials, and number of psychotropic medication trials. However, the group-by-time interaction remained nonsignificant.

### Protocol Completers

The overall number of patients achieving remission from suicidality, as indexed by the Beck SSI score, was 21 of 36 protocol completers (58.3%). The overall mean (SD) Beck SSI score decreased from 10.6 (4.6) at baseline to 4.8 (6.4) at the end of the trial (paired *t*_35_ = 2.03; *P* < .001) (Figure 3). An analysis similar to the aforementioned analysis with the suicide item of the HRSD-24 validated these results with qualitatively similar outcomes (data not shown). The odds of remission from suicidality were lower in the adequate trial completer group compared with the protocol completers group, although the difference was not significant (odds ratio, 0.65; 95% CI, 0.29-1.48). For the subgroup sensitivity analysis, the participants treated with low- and moderate-frequency MST had numerically higher rates of remission from suicidality (11 participants [73.3%] and 7 participants [58.3%], respectively) than those treated with high-frequency MST (3 participants [33.3%]) (Figure 2); however, the differences between these rates were not statistically significant (low vs high frequency, χ^2^_1_ = 3.70; *P* = .05).

## Discussion

In this secondary analysis of an open-label trial of MST for TRD, we explored the association of MST with suicidality. Overall, MST treatment was associated with reductions in suicidality that were both statistically significant and clinically meaningful in both the adequate trial group and per protocol treatment subgroup. The findings with our primary outcome measure (the Beck SSI score) were confirmed by additional analyses with the HRSD-24 suicide item, even though these 2 measures were only moderately correlated in our study. Our sensitivity analysis revealed that all MST treatment frequencies were associated with a reduction of suicidality, and that low- and moderate-frequency MST were associated with the highest rates of remission clinically from suicidality in both the group of participants who completed an adequate MST trial (55.2% for low-frequency MST and 54.5% for moderate-frequency MST) and in the subgroup who completed the protocol (73.3% for low-frequency MST and 58.3% for moderate-frequency MST). However, the differences between these rates of remission from suicidality were not significant, possibly because of the small sample sizes of the 3 groups.

Our results are exploratory and represent a post hoc analysis, so comparison with established treatments is limited. Nonetheless, we attempt to give some sense of comparative efficacy to other brain stimulation treatments used for suicidality in TRD. In the largest study of ECT and suicidality in depression, Fink et al.^11^ measured suicidality with the HRSD-24 suicide item, pooling data from 2 ECT trials^13,30^; they reported a rate of remission from suicidality of 63.2%. This is comparable to the rate of remission from suicidality of 47.8% that we observed in our primary analysis for MST. The differences in the methods in that ECT study and our MST study demand a direct comparison in a randomized study, which is currently under way (ClinicalTrials.gov Identifier: NCT03191058). Considering that early evidence suggests that MST can be delivered with far less stigma and fewer cognitive adverse effects than ECT,^22^ MST may be a preferred treatment for suicidality in many patients. The rate of remission from suicidality we observed with MST also suggests potential superiority of MST over rTMS. We previously described the association of bilateral rTMS with remission from suicidality as measured with the HRSD-24 suicide item at 40.4% with bilateral rTMS^17^ (compared with 47.8% in the current study). Again, differences in study methods here greatly limit this interpretation, and direct comparisons should be performed in future studies.

Some of our own and others’ recent work sheds light on potential mechanisms of action of MST in treating suicidality, and why low and moderate frequencies could be the most effective treatment frequencies. Backhouse et al^31^ reported that seizure adequacy (a composite measure of several seizure characteristics) was better in patients with TRD receiving 25-Hz and 50-Hz MST than in those receiving 100-Hz MST. This may be, in part, due to 100-Hz treatment not entraining pyramidal cells optimally, because this frequency is too high given the duration of the refractory period of cortical neurons, thereby inhibiting optimal recurrence of neuron depolarization (ie, seizure activity).^31^ This hypothesis should be evaluated in future MST trials, because it is still uncertain how to optimize treatment parameters for each individual patient receiving MST.^32^ In 2 earlier, overlapping studies,^33,34^ we found that transcranial magnetic stimulation–electroencephalographic measures, such as long-interval cortical inhibition and N100, could be used to estimate improvement in suicidality in patients with TRD treated with MST. In the earlier of the 2 analyses,^33^ improvement in suicidality appeared to be mediated by dorsolateral prefrontal cortex plasticity through changes of GABAergic activity. It is possible that GABA-mediated plasticity in the dorsolateral prefrontal cortex was associated with the antisuicidal association of MST in our participants. MST may also be associated with reductions in suicidality through its action on other prefrontal brain areas, potentially medially located regions such as the dorsomedial prefrontal or ventromedial prefrontal cortices, or possibly the frontopolar cortex.^35^ These regions are not targeted with traditional rTMS, which could account for potential differences between the associations of MST and rTMS with suicidality. Although these mechanisms are possible, other studies have emphasized the role of alternative neurobiological mechanisms involving the hypothalamic-pituitary-adrenal axis, serotonin system, or glutamate and opioid signaling in suicidality, which could be targeted by MST.^36^

Overall, changes in depressive symptoms were only moderately correlated with changes in suicidality in our adequate trial completers (data not shown). Also, in an unpublished analysis of the factors associated with depression remission from MST in the larger sample of 86 suicidal and nonsuicidal participants, higher baseline suicidality was associated with lower odds of depression remission (D.M.B., J.D., Z.J.D., unpublished data, 2019). Taken together, these results support the clinical wisdom that change in suicidality is not necessarily congruent with change in overall depression. This furthers the notion of suicidality being its own neuro-endophenotype and symptom construct independent of, yet highly comorbid with, other psychiatric illness,^36,37,38^ which should be explored further in future clinical trials.

### Limitations

There are multiple limitations to this study. Although, to our knowledge, this is the largest trial to date to evaluate the association of MST with suicidality in TRD, it is open label, and participants were assigned sequentially to different treatment frequencies. As such, there is the potential for bias, with treaters and participants being aware of the experimental nature of the treatment and of the frequency of MST received. Also, a non-MST comparator group (eg, placebo or another active treatment) was not used. There is also the risk of false-positive results with the multiple statistical tests in this report. Patients with a suicide attempt during the preceding 6 months or those at risk for an imminent suicide attempt were excluded, thus limiting application of our findings to these types of patients. Similarly, given our exclusion criteria, our results may not be directly applicable to patients with borderline personality disorder, bipolar illness, substance use disorders, or other disorders with high rates of suicidality. In addition, although we did find clinically meaningful differences in response by treatment frequency, we highlight that these differences were not statistically significant, likely because of the small sample sizes.

## Conclusions

MST appears to be a promising intervention to target suicidality. Its efficacy will need to be confirmed in future MST trials exploring its use, potentially at low-to-moderate frequencies to start, in patients experiencing suicidality. A direct comparison is also needed to determine the relative associations of MST and ECT with suicidality. Such a comparison is currently under way.

## Notes

Supplement. **eTable. **Suicidality Scores by Treatment Frequency for Adequate Trial Completers **eAppendix.** MST Detailed Project Protocol **eReferences**
