# Supplementary material for: Magnetic Seizure Therapy for Suicidality in Treatment-Resistant Depression
Source: JAMA Netw Open. 2020 Aug 18;3(8):e207434. doi: 10.1001/jamanetworkopen.2020.7434 (PMC7435344; doi:10.1001/jamanetworkopen.2020.7434)
Supplement: Supplement. — eTable. Suicidality Scores by Treatment Frequency for Adequate Trial Completers eAppendix. MST Detailed Project Protocol eReferences [file jamanetwopen-3-e207434-s001.pdf]

Supplementary Online Content

Weissman CR, Blumberger DM, Dimitrova J, et al. Magnetic seizure therapy for suicidality in treatment-resistant depression. *JAMA Netw Open*. 2020;3(8):e207434. doi:10.1001/jamanetworkopen.2020.7434

eTable. Suicidality Scores by Treatment Frequency for Adequate Trial Completers
eAppendix. MST Detailed Project Protocol
eReferences

This supplementary material has been provided by the authors to give readers additional information about their work.

© 2020 Weissman CR et al. *JAMA Network Open.*

eTable. Suicidality Scores by Treatment Frequency for Adequate Trial Completers[a]

	Treatment Frequency		
	**Low frequency** n=29	**Moderate** n=22	**High** n=16
**HRSD suicide item at baseline**	1.6 (0.8)	1.4 (0.6)	2.6 (0.8)
**HRSD suicide item at endpoint**	0.9 (1.0)	1.0 (0.9)	1.5 (1.3)
**HRSD relative reduction (%)**	43.6	33.3	41.7
**Rate of remission from suicidality[b] (%)**	40.0	36.8	28.6

All results are presented as mean (SD) unless indicated otherwise.
HRSD: Hamilton Rating Scale for Depression
a Adequate trial completers: participants completing eight or more MST sessions
b Remission from suicidality defined as a final score of 0

eAppendix. MST Detailed Project Protocol

Background

Treatment Resistant Depression and Electroconvulsive Therapy

Depression is a common (~15-20% life prevalence) and important clinical problem with high morbidity and mortality. A significant subset of depressed patients continue to experience highly distressing and disabling symptoms despite standard treatments [1]. This subset has been estimated to be in the range of between 10 and 20% of patients with the disorder [1]. Electroconvulsive therapy (ECT) is the most effective treatment for major depressive disorder [2]. However, many patients are reluctant to engage in a trial due to stigma and the risk of cognitive side effects [3] The development of cognitive impairment, particularly memory impairment, is a particularly troubling side effect of ECT and often leads to treatment non acceptance. Anecdotally, many patients who have had successful ECT will not return for follow-up treatment upon the relapse of the depression because of such side effects. Modifications to the ECT treatment procedure that could substantially reduce or minimize these side-effects would, therefore, be of substantial benefit. Importantly, both the efficacy and side effects produced by ECT may be affected by a variety of treatment parameters such as electrode placement, electrical dose and also potentially the pattern of seizure initiation and spread [4]. However, the degree to which these factors can be varied is limited in ECT, particularly with regard to the way in which the seizure is initiated [4].

Treatment-Resistant Schizophrenia and Electroconvulsive Therapy

Schizophrenia (SCZ) is a debilitating disorder that exacts enormous personal, social and economic costs. Despite recent advances in psychopharmacological treatments nearly 40% of patients achieve only a partial response and 10% experience no response at all [5]. To date, only a few alternatives have been available: these generally include clozapine and ECT. Both, however, are associated with significant side effects. For example, clozapine has been associated with hyperlipidemia, blood dyscrasias, diabetes, seizures and cardiomyopathy [6,7]. Furthermore, some patients will not tolerate the rigorous monitoring associated with clozapine treatment. Similarly, ECT is associated with significant cognitive impairment and the stigma associated with ECT limits its broader use as a refractory treatment. Together, these limitations highlight the need for additional treatments aimed at ameliorating the sequelae of SCZ. To date, no studies have reported the use of magnetic seizure therapy (MST) in a population of patients with refractory SCZ.

Treatment Resistant Obsessive Compulsive Disorder and Electroconvulsive Therapy

© 2020 Weissman CR et al. *JAMA Network Open.*

Obsessive compulsive disorder (OCD) is a prevalent and highly debilitating illness. Estimates suggest that close to 3% of the general population have this illness[8]. Despite advances in psychotherapeutic and psychopharmacological treatments, approximately 50% of patients with OCD remain refractory to treatment[9]. ECT is indicated for refractory OCD, yet the data is mainly from case series[10]. Other non-pharmacologic treatments include implantable deep brain stimulation and neurosurgical ablation which carry significant risk of morbidity and side effects. Due to the high rates of treatment resistance and the severe morbidity of the illness, treatment alternatives are required. MST holds the potential to be another potential treatment for treatment refractory OCD. To date, no studies of MST in OCD have been conducted

Magnetic seizure therapy

Like ECT, MST involves the intentional induction of a seizure for therapeutic purposes. However, the induction of a seizure occurs through the use of high frequency repetitive transcranial magnetic stimulation (rTMS) rather than through stimulation of the brain with a direct electrical current, such as that which occurs with ECT. In this context, MST should be differentiated from rTMS, which uses the same or similar equipment but is non convulsive. rTMS involves the application of magnetic pulses at a considerably lower intensity and frequency. It is likely that rTMS and MST have substantially differing mechanisms of action.

As has been practised to date, MST is administered under general anesthesia in much the same way as ECT. MST, like rTMS, does not involve the direct application of electricity. Instead, electrical current is produced indirectly in the brain via electromagnetic induction. The rTMS stimulator induces a high field magnetic pulse, which passes into the brain, inducing an electrical current in brain cells. This capacity to indirectly stimulate the brain overcomes one of the major problems with ECT: its inability to provide focal or directed stimulation. Although a number approaches have tried to apply ECT in focused ways (e.g frontal, temporal), the substantial electrical impedance of the scalp and skull means that the bulk of the electrical stimulus is shunted away from the brain, resulting in stimulation of widespread cortical and subcortical regions [4]. However, as there is no resistance to the passage of the magnetic field produced by an rTMS device into the brain, magnetic stimulation may be focused quite precisely. Therefore, it is possible that seizures may be produced with less spread to medial temporal lobe structures, reducing memory related side-effects [11].

There is another substantial difference in regards to the mechanism of seizure induction between MST and ECT related to pulse width. Basic physiological studies have suggested that the best pulse width for stimulating cortical neurons may be briefer than that used in ECT. Briefer pulses excite neurons more efficiently at lower charge densities and have a larger safety margin because of their lower charge per phase [12]. Recent ECT studies have investigated briefer pulse width stimulation to try and reduce cognitive side effects [13,14]. The pulse width of an rTMS stimulator (typically 0.2ms) is in the ultra brief range, which might be expected to enhance its efficacy of seizure induction, although the waveform itself differs between magnetic stimulation and ECT. Due to differences in pulse wave form, a lengthening of the MST pulse to 0.5 ms has been found to be more effective [15].

Animal Studies

The initial research with MST was conducted in nonhuman primates with the first MST- induced seizure reported in 1998 [15]. Custom modified rTMS devices capable of stimulating at higher intensities and frequencies were developed for this purpose. Several important outcomes of this research have emerged. First, MST has been shown to not produce identifiable histological lesions in the brain in primates [16]. Second, information with regard to optimal stimulation parameters for seizure induction has been gathered [15]. Third, there appears to be fewer cognitive side effects with MST, as opposed to ECT, in this animal model [17].

Human Studies

© 2020 Weissman CR et al. *JAMA Network Open.*

In parallel, a number of initial human studies have been undertaken. The first patient received MST stimulation over 4 sessions [15] and a second successfully received a full treatment course [18]. Both patients were treated with stimulation at 40 Hz, tolerated the treatment well and responded clinically. In a subsequent study, 10 patients received two MST sessions within a course of ECT [19]. The MST sessions were better tolerated and resulted in fewer acute side-effects compared to the ECT. Notably, in 3 of the 10 patients, the MST seizure threshold was at the maximum output of the device. 20 patients were subsequently treated with a full course of MST using the same 50 Hz device [19,20]. Mood improvement was again seen in the MST group with fewer side effects and dramatically more rapid reorientation post-stimulation than in ECT group. However, the magnitude of improvement did not seem as great as that of ECT. The authors have speculated that, as the stimulation dose was, on average, only 1.3 times the magnetic seizure threshold, substantially greater responses may have been achieved with higher stimulation intensity, especially given that ECT response rates are highly sensitive to dose relative to seizure threshold [21].

Since these initial studies, the technology used to produce MST has advanced considerably. Two companies have developed MST devices capable of stimulating continuously at 100Hz for sufficient durations to induce seizures. This type of stimulation has been shown in primate experiments to induce seizures and still demonstrates fewer cognitive side-effects than conventional ECT [22]. This type of stimulation has now been tested in human subjects, with 11 patients being stimulated with 100 Hz MST in a single session during a regular course of ECT. Seizures were elicited in 10 of the 11 patients. All patients had a highly rapid recovery of orientation (on average 15 minutes shorter than recovery time after ECT) and reported less confusion [23].

In addition to the published reports, we are aware of studies underway utilizing in MST in a number of centres around the world. Four centres (Columbia University, the University of Texas Southwestern Medical Centre (US), Cardiff University and the University of Oxford (UK)) are currently using an MST device produced by Magstim (Whitland, UK). Two centres in Germany (Bonn and Berlin), as well as the centre at Columbia University have commenced studies with the device relevant to this application, the Magventure A/S MST Magpro MST device.

Summary

Treatment resistant depression, schizophrenia and OCD remain substantial clinical problems for which there are limited alternatives. MST is a novel modification of ECT with the potential for similar effectiveness, fewer side-effects and a more rapid return of orientation / shorter duration of post-ictal confusion. Furthermore, MST is not associated with the same cognitive burden and media-related stigma as ECT, which may lead to broader acceptance if it is found to be effective. However, experience with this technique has been limited to date and considerable further research is required. In this application we propose to conduct an initial Canadian pilot study of MST using a stimulator capable of 100 Hz. Within this pilot study, patients will be treated in an open label manner to observe response rates, cognitive implications of MST treatment as well as several neurobiological variables that may enhance our understanding of treatment response. The results of the study should allow us to proceed to a double-blind randomized comparison of MST with ECT.

Methods

Objectives and hypotheses

Objective 1: To evaluate the efficacy of MST in with severe depression, schizophrenia, and OCD.
Hypothesis 1: MST will demonstrate substantial efficacy (i.e., substantial rates of response and remission) on objective measures of mood, schizophrenia, and OCD symptoms.
Objective 2: To evaluate the effects of MST on autobiographical memory and other cognitive functions in patients with severe depression, schizophrenia, and OCD.
Hypothesis 2: MST will have limited adverse effects on objective measures of autobiographical memory and other cognitive functions in patients with severe depression, schizophrenia, and OCD.
Objective 3: To compare the changes in brain function that result from MST.

Hypothesis 3: MST will produce changes in functional brain activity consistent with antidepressant response, antipsychotic response, and antiobsessive response, along with a sparing of cognitive functions.

Design

The core study will involve an open label design with before-, during- and after- treatment assessments of depression severity, subjective side-effects and cognitive performance. We will assess baseline treatment resistance using the antidepressant treatment history form (ATHF) [24]. Baseline medical comorbidity will be assessed using the Cumulative Illness Rating Scale(CIRS)[25]. Cognition will be monitored at baseline, at every 6 treatments, and at the end of the treatment course. In addition, we will engage patients in pre- and post- treatment neuroimaging and cortical inhibition measures to study the biological effects of MST treatment.

Subjects

A total of 250 patients will be included in the study, including at least 20 schizophrenia and 20 OCD patients, 20 with major depressive episode with psychotic features in the context of Major Depressive Disorder or Bipolar Disorder, 150 major depressive episode without psychotic features in the context of Major Depressive Disorder, 40 major depressive episode without psychotic features in the context of Bipolar Disorder. With a 10 point difference in mean pre – post 24-item HDRS scores and a standard deviation of 8, the smallest group (n=11) in this sample should still have a power approaching 1 (>0.99) (alpha =0.05, 2 tailed).

Inclusion Criteria:

Patients will be included if they:

1. have a DSM-IV diagnosis of a major depressive episode with or without psychotic features in the context of major depressive disorder or bipolar disorder, obsessive compulsive disorder, or schizophrenia/schizoaffective based on SCID-IV criteria
2. are within the age range from 18-85
3. have a 24-item HDRS score of $\geq$ 21 (depression patients, moderate – severe)
4. have an 18-item BPRS score of $\geq$ 37 (schizophrenia/schizoaffective patients, moderate – severe)
5. have a Y-BOCS score of $\geq$ 16 (OCD patients, moderate – severe)
6. demonstrates capacity to consent according to study and treating psychiatrist or MacCAT for subjects with schizophrenia/schizoaffective
7. are on a medically acceptable form of birth control, if a woman of child-bearing potential

Exclusion Criteria:

Patients are excluded if they:

1. have an unstable medical and/or neurological condition
2. are currently pregnant or lactating
3. are not considered sufficiently well to undergo general anesthesia for any reason
4. have a cardiac pacemaker, cochlear implant, implanted electronic device or non-electric ferrometallic implant in the head only
5. are taking a benzodiazepine at a dose greater than lorazepam 2mg or equivalent
6. are taking any non-benzodiazepine anticonvulsant
7. have active substance misuse or dependence within the past 3 months
8. have a current diagnosis of delirium, dementia or another cognitive disorder secondary to a general medical condition
9. have other significant Axis I co-morbidity
10. have a co-morbid borderline personality disorder and/or antisocial personality disorder as confirmed by the Structured Clinical Interview for DSM-IV Axis II Disorders (SCID-II)
11. have had a history of any suicide attempts in the past 6 months

Clinical Measures

Demographic variables and potential co-variates will be recorded at baseline following a clinical interview. These will include the duration of the current episode, years from first diagnosis, number of previous episodes, type and dose of current and previous treatment and family history of mood disorder. Diagnosis will be assessed with the SCID (DSM IV). Clinical rating measures will include the 24-item Hamilton

© 2020 Weissman CR et al. *JAMA Network Open.*

Depression Rating scale (HDRS) for consistency with most prior ECT studies, as well as the Quick Inventory of Depressive Symptomatology (QIDS) and Brief Psychiatric Rating Scale (BPRS) and YBOCS (Yale-Brown Obsessive-Compulsive Scale), as appropriate. For those patients with Major Depressive Disorder and Bipolar Disorder we will use the Young Mania Rating Scale (YMARS) to monitor for emergence of hypomania. Given the significant toll of these illnesses on quality of life, all patients will answer the Quality of Life Enjoyment and Satisfaction Questionnaire (Q-LES-Q)[50] prior to and at the end of the acute treatment phase. The Beck Scale for Suicide Ideation (BSS)[27][26] will be used to evaluate suicidal ideation, which is a common symptom in depression. A systematic evaluation of suicidal ideation is needed in any depression treatment study for safety reasons and because clinicians need to know the effects of treatment on suicidality to understand risks vs. benefits. At each clinical assessment if score on the BSS is ≥ 9 the patient will have a consultation with a study psychiatrist to address any passive or active suicidal ideation. The Snaith-Hamilton Pleasure Scale (SHAPS) will be used to evaluate and monitor anhedonia in participants with depression and the effects of treatment on this core feature of depression [55].

The entire clinical battery will be repeated after every three treatments, at the end of acute treatment phase, and at 1, 2, 3, and 6 months after the acute treatment phase. The battery will again be repeated 6 months after the maintenance MST treatment phase, if applicable. The QIDS will be administered prior to every treatment visit for those subjects with depression in order to check for symptom improvement between clinical assessments. If the score falls within the euthymic range (0-5), the entire clinical battery will be administered to assess possible remission. For depression, remission will be defined as a 24-item HDRS ≤ 10, and a greater than 60% decrease in scores from baseline on two consecutive ratings with a change no greater than 3 points. Response criteria will be defined as a ≥50% reduction in 24-item HDRS score from baseline on two consecutive ratings. For schizophrenia, response will be defined as an 18-item BPRS < 25, for consistency of criterion with recent large studies of ECT in treatment-resistant schizophrenia[27]. For OCD, remission will be defined as a Y-BOCS score ≤8, and a greater than 60% decrease in scores from baseline on two consecutive ratings with a change no greater than 3 points.

Cognitive assessment
All patients will be assessed with the entire cognitive battery prior to and at the end of the acute treatment phase, and at 6 months post-treatment. The MOCA will be administered at baseline and every 6 treatments during acute treatment. The MOCA will also be administered before the last treatment if the patient will not be receiving maintenance treatment, and will be done before their first maintenance treatment if continuing onto the maintenance phase. It will then be administered once a month during maintenance treatment. The cognitive battery will include assessments of anterograde and retrograde memory, specifically looking at learning, retention and retrieval in both the verbal and non verbal domains. This will include assessments such as the Autobiographical Memory Interview Short Form[28], MATRICS Consensus Cognitive Battery (MCCB), Stroop and Verbal Fluency using the COWAT . Finally we will look at general intellectual functioning with the WTAR (Wechsler Test of Adult Reading) prior to treatment start. Cognitive function during the treatment phase of the study will be assessed with the Montreal Cognitive Assessment (MoCA). This test has three different English versions which allow us to avoid practice effects.

Additionally, time to reorientation will also be measured after each MST session using previously published standardized methods [30,31] that evaluate orientation to name, date of birth, age, place and day of the week. This will be accomplished by repeatedly asking orientation questions from the time of resumed respiration post-MST and noting the recovery time required to recall 4 of the 5 items listed above [32].

MST treatment procedure
The range of frequency stimulation used to treat patients will be between 20 Hz and 100Hz, with a duration range between 2 and 20 seconds depending on the frequency used. Furthermore, we would also like to specify that the anatomical location of stimulation will be either the frontal or vertex region of the brain. These are the two sites at which stimulation is most commonly applied [32, 56, 57]. The MST determination of seizure threshold will be done at 100% stimulator output applied at the selected treatment frequency with progressively escalating train durations until an adequate seizure is produced. During an

© 2020 Weissman CR et al. *JAMA Network Open.*

ECT treatment an adequate seizure is described as generalized tonic-clonic activity $\geq$ 20 seconds on the EMG recording or $\geq$ 25 seconds of EEG seizure activity. However, little data is available on the characteristics of MST induced seizures therefore the adequacy of the seizure will be determined at each session by the treating MST psychiatrist. During titration a maximum of three stimulations will be given at the same session, provided the coil temperature allows for a third stimulation. If an adequate seizure is not produced by the third stimulation, titration will continue at the next treatment session until threshold is reached. Subsequent treatments will then be delivered according to the established conventions for delivering MST.

Acute Treatment Phase

Six treatment sessions, at a frequency of two or three times per week will be administered. Patients will not have treatments on consecutive days. As previously stated, remission will be assessed at every 3 treatments, and if the pre-defined remission rate is not met 3 additional treatments will be provided. This will be repeated a total of 5 times (i.e., maximum treatment number is 24). 24 treatments is typically longer that a conventional ECT treatment course. However, evidence does suggest that longer treatment courses may be needed with MST, particularly in more treatment resistant psychiatric conditions such as OCD and Schizophrenia [51-52]. Furthermore, response during the acute treatment course will also be monitored and the dose adjusted accordingly. In the study the dose was originally adjusted at every 6 sessions. However, response during the acute treatment course will also be monitored and the dose adjusted accordingly every three sessions rather than every 6 sessions. That is, if the patient fails to achieve an equal or greater than 30% decrease from baseline following treatment 3, the dose will be increased on their 4th treatment. After treatment 6, if the patient fails to achieve further response, that is an equal or greater than 30% decrease from the score after treatment 3, the dose will be increased on their 7th treatment. Treatment continues in this manner up to a maximum of 24 total treatments. If at any point the patient is already at maximum stimulation (20 seconds or 1000 pulses) the treatment continues with the dose unchanged.

Patients will be withdrawn from the study and offered to switch to ECT if, despite MST treatment, they experience a significant clinical decline or an acute worsening of symptoms that creates concerns over their safety. Specific criteria for withdrawal from MST treatment would include 1) the emergence of suicidal intent or plan; 2) suicide attempt; 3) serious attempt to harm others; 4) emergence of catatonia; 5) emergence of severe inanition (e.g., failure to consume food or fluids). Any incidents of safety concerns requiring a switch to ECT will be monitored as one of the MST efficacy measures in the study. Non-responding subjects can be withdrawn from the study prematurely if the investigator deems this to be in the patient's best interest. Additionally, subjects will be discontinued from the study if more than 3 consecutive treatments are missed.

Maintenance Treatment Phase

Rates of depressive relapse after a successful course of ECT can be as high as 50% within the first 6 months [53]. Similarly, psychotic exacerbation can occur at high rates after patients with schizophrenia improve with ECT [51]. Evidence based strategies to prevent depressive relapse within the first 6 months after a successful course of ECT include the combination of an antidepressant and lithium or maintenance ECT [53, 54]. Maintenance ECT is often used after patients with schizophrenia improve with ECT [51]. There is very little data on rates of relapse after a successful course of ECT in patients with OCD; nevertheless, there is no reason to believe that it would not have a similarly beneficial effect at reducing relapse. To date, there have not been any reports on the use of maintenance MST. Therefore, all patients who attain the a-priori defined remission and/or response criteria in the acute treatment phase of MST will be offered participation in maintenance MST as means of preventing relapse. Subjects will also be provided with psycho-education and recommendations will be made to their attending physicians regarding alternative evidence based treatments to prevent relapse. Those subjects that elect to receive maintenance MST will receive treatment according to the standard maintenance schedule used for ECT: one treatment per week for 4 weeks, then one treatment every two weeks for 2 months, then one treatment every 3 weeks for 2 months and then one treatment 4 weeks later. Subjects will be assessed at regularly scheduled intervals throughout the maintenance phase and as needed, to determine if they are maintaining response or remission. If subjects have two consecutive scores above the a-priori defined remission or response score they will be offered two weeks of booster treatments (2 treatments per week). If they do not respond after two weeks they will exit from the study and alternative treatment will be discussed clinically. If the subject

does not wish to receive booster treatments they will exit from the study and alternative treatment will be discussed clinically.

Safety Considerations
MST is an involved treatment, with many congruencies to ECT in regards to the short-term side effects experienced following treatment session(s). As mentioned, MST has been shown to result in less cognitive burden than treatment with ECT. However, there are other potential side effects that we anticipate over the course of the trial. This section discusses anticipated adverse events based on a careful review of existing research literature regarding ECT and MST treatment. This review has been further supplemented by subject reports received to date.

The following adverse events are anticipated in a sub-sample of the participant population: reversible cardiac ectopy, transient hypertension, uncomplicated asystole, fatigue, headache, aching/stiffness in muscles, nausea and vomiting, acute post-treatment delirium, post-ictal agitation, disorientation, memory impairment (e.g., anterograde and retrograde memory loss), prolonged seizures (i.e., seizures > 120 seconds in duration), treatment emergent mania, treatment emergent anxiety and fear, laryngospasm, peripheral nerve palsies, and aspiration.

Several steps are taken to mitigate the risk of side effects. Prior to treatment all MST patients at CAMH receive an in-depth consultation from an ECT psychiatrist. The purpose of the consultation is to assess illness type and severity, previous treatments and outcomes, relative contraindications, discuss risks and benefits, and capacity to consent to ECT/MST. In addition to the psychiatric consult, all potential subjects receive a pre-MST consultation from the anesthesia service to assess suitability for general anesthesia, medical comorbidities that may impact anesthesia, discuss the risks of general anesthesia, and finally, conduct the informed consent for general anesthesia. During all treatment sessions, vital signs (heart rate, BP, O2 saturation, ECG, EEG) are monitored continuously. Patients undergo preoxygenation, anesthesia and muscle relaxation with accepted medications used in ECT practice, bite guard placement, and immmoblization prior to seizure induction. After EEG-confirmed seizure termination and recovery from anesthesia, patients are transferred to a recovery room once vital signs are stable and breathing.

In the event a subject experiences a side effect, several steps are taken to minimize discomfort. Additional medications for symptomatic relief of side effects will be used based on accepted medications used in ECT practice For example, participants reporting moderate to severe headaches during previous treatments may receive an anti-inflammatory medication prior to their next treatment as a means of preventing headaches. Similarly, a participant that experienced nausea or vomiting will be given an intravenous anti-nausea medication to ease symptoms at the next treatment. Participants are also encouraged to take Tylenol for any muscle soreness. Risks of wakening paralysis and post-ictal agitation are mitigated by giving midazolam post-treatment. This is done on a case-by-case basis after careful observation of the patient's reaction to the first treatment.

Analysis of Clinical Outcomes
Depression scores and cognitive performance will be compared pre- and post-treatment using paired t-tests, with significance thresholds Bonferroni-corrected for multiple comparisons.

Biomarkers of Treatment Response
Participation in this component of the protocol is voluntary does not preclude participation in the treatment component of the trial.

Changes in Cortical Inhibition
Dysfunctional cortical inhibition (CI) has been postulated as a mechanism through which the symptoms of MDD, SCZ and OCD are mediated [33-36]. Cortical inhibition refers to the neurophysiological process in which γ-aminobutyric acid (GABA) inhibitory interneurons selectively attenuate the activity of pyramidal neurons in the cortex. TMS capitalizes on the ability of time-varying magnetic fields to induce eddy currents in biological tissue via the principle of electromagnetic induction. TMS can provide an index of

© 2020 Weissman CR et al. *JAMA Network Open.*

GABA receptor-mediated inhibition in the cortex because it differentially stimulates inhibitory interneurons and pyramidal neurons. There are several TMS paradigms which provide a measure of GABA receptor-mediated inhibitory neurotransmission long interval cortical inhibition (LICI) [37-41], the cortical silent period (CSP) and short-interval cortical inhibition (SICI). Single or paired-pulse transcranial magnetic stimulation (TMS) represents a unique experimental modality that has been used to directly index CI. With single and paired-pulse TMS we have recently demonstrated that MDD is associated with deficits in CI that are more pronounced in patients with SCZ, OCD and MDD. It has also been shown that treatment with electroconvulsive therapy (ECT) is associated with enhanced CI [35,42-44]. Collectively, these studies suggest that potentiation of CI may represent a unique neurophysiological mechanism through which brain stimulation treatments (i.e., ECT and MST) exert their therapeutic effects in the above mentioned psychiatric disorders.

TMS shall be used to examine GABAB receptor mediated inhibitory neurotransmission, through LICI, in both the DLPFC and motor cortex. Further, we will also evaluate the CSP in the motor cortex as a second index of GABAB receptor mediated inhibitory neurotransmission in the motor cortex. Evaluation of the DLPFC and motor cortex will be conducted in counterbalanced order and each region will be assessed immediately after completion of the other. To evaluate LICI from the DLPFC, EEG recordings will be acquired through a 64-channel Synamps 2 EEG system. Electrodes of interest will include those which optimally represent the overlap of Brodmann areas (BA) 9 and 46 of the DLPFC that has been shown to be most closely associated with electrode AF3. All neurophysiological data will be coded and analysed by an experienced rater. Measures of cortical inhibition will be administered twice, prior to and at the end of the acute treatment phase within 48hrs of the first and last MST treatment.

Measurement of Working Memory

As one of the main objectives of this study is to evaluate the effects of MST on autobiographical memory and other cognitive functions, we will also administer a working memory task prior to and at the end of the acute treatment phase. Working memory will be examined with the verbal N-back task (1- and 3-Back task) while EEG is recorded at the same session as TMS-EEG analysis at baseline and following an acute course of MST treatment. This test will allow us to further explore the cognitive affect of MST as well as examine the relationship between working memory and cortical inhibition.

Neuroimaging

In addition to measures of cortical inhibition, all patients will also undergo three sessions of neuroimaging: one prior to treatment, one within 48 hours of the end of treatment, and one at 6 months after treatment. Each session will include 3 types of neuroimaging studies in order to assess changes in brain structure and brain function, as follows:

1. A T1-weighted MRI sequence to provide high-resolution anatomical images of the cerebral gray and white matter. These images will be used for anatomical co-registration of the functional neuroimaging data, and for voxel-based morphometry (VBM) to assess regional changes in cortical and hippocampal gray matter thickness before and after treatment. Pre- and post-treatment scans will be compared using standard SPM8 and FSL software for identifying statistically significant regional changes in whole-brain VBM data.

2. A diffusion tensor imaging (DTI) MRI sequence to provide detailed information on the structural integrity of white matter tracts in the prefrontal lobes, hippocampus, and other cortical regions before and after treatment. Pre- and post-treatment scans will be compared using standard FSL software for reconstruction and statistical comparison of white matter tract structure, trajectory, and integrity.

3. A T2*-weighted, functional MRI (fMRI) sequence to assess blood oxygenation level dependent (BOLD) signal changes marking increases and decreases in regional functional connectivity to the amygdala and ventral striatum during the resting state, before and after treatment. Pre- and post treatment scans will be analysed using standard SPM8 and FSL software for identifying statistically significant regional changes in whole-brain BOLD signal data.

© 2020 Weissman CR et al. *JAMA Network Open.*

The first aim of the neuroimaging measures is to determine whether MST results in changes in brain structure and activity that are consistent with antidepressant response to other treatments, including ECT, DBS, and antidepressant medications. Based on previous studies, we propose that this will include increases in resting brain metabolism on PET in dorsal prefrontal and dorsal anterior cingulate cortex and decreases in orbitofrontal prefrontal cortex and ventral striatum [29-31]. The second aim of the neuroimaging measures is to determine whether MST also has milder effects on the brain structures most closely linked to episodic memory and other related cognitive functions. We also propose that there will be a more pronounced recovery of functional activity in the hippocampal and retrosplenial regions on PET and fMRI measures[31], as well as volumetric expansion in the hippocampus on VBM[32], and improved hippocampal-prefrontal and hippocampal-retrosplenial white matter tract integrity on DTI measures[33].

© 2020 Weissman CR et al. *JAMA Network Open.*

eReferences

1. Fava M. Diagnosis and definition of treatment-resistant depression. *Biol Psychiatry.* 2003;53(8):649-659.
2. Ebmeier KP, Donaghey C, Steele JD. Recent developments and current controversies in depression. *Lancet.* 2006;367(9505):153-167.
3. Lisanby SH. Electroconvulsive therapy for depression. *N Engl J Med.* 2007;357(19):1939-1945.
4. Lisanby SH. Update on magnetic seizure therapy: a novel form of convulsive therapy. *J ECT.* 2002;18(4):182-188.
5. Pantelis C, Lambert TJ. Managing patients with "treatment-resistant" schizophrenia. *Med J Aust.* 2003;178 Suppl:S62-66.
6. Sernyak MJ, Leslie DL, Alarcon RD, Losonczy MF, Rosenheck R. Association of diabetes mellitus with use of atypical neuroleptics in the treatment of schizophrenia. *Am J Psychiatry.* 2002;159(4):561-566.
7. Iqbal MM, Rahman A, Husain Z, Mahmud SZ, Ryan WG, Feldman JM. Clozapine: a clinical review of adverse effects and management. *Ann Clin Psychiatry.* 2003;15(1):33-48.
8. Weissman MM, Bland RC, Canino GJ, et al. The cross national epidemiology of obsessive compulsive disorder. The Cross National Collaborative Group. *J Clin Psychiatry.* 1994;55 Suppl:5-10.
9. Kaplan A, Hollander E. A review of pharmacologic treatments for obsessive-compulsive disorder. *Psychiatr Serv.* 2003;54(8):1111-1118.
10. Dell'Osso B, Altamura AC, Allen A, Hollander E. Brain stimulation techniques in the treatment of obsessive-compulsive disorder: current and future directions. *CNS Spectr.* 2005;10(12):966-979, 983.
11. Sackeim HA, Long J, Luber B, et al. Physical properties and quantification of the ECT stimulus: I. Basic principles. *Convuls Ther.* 1994;10(2):93-123.
12. McCreery DB, Agnew WF, Yuen TG, Bullara L. Charge density and charge per phase as cofactors in neural injury induced by electrical stimulation. *IEEE Trans Biomed Eng.* 1990;37(10):996-1001.
13. Kim SW, Grant JE, Rittberg BR, Simon JE, Vine CJ, Schulz SC. Decreased memory loss associated with right unilateral ultra-brief pulse wave ECT. *Minn Med.* 2007;90(1):34-35.
14. Sackeim HA, Prudic J, Nobler MS, et al. Effects of pulse width and electrode placement on the efficacy and cognitive effects of electroconvulsive therapy. *Brain Stimul.* 2008;1(2):71-83.
15. Lisanby SH, Schlaepfer TE, Fisch HU, Sackeim HA. Magnetic seizure therapy of major depression. *Arch Gen Psychiatry.* 2001;58(3):303-305.
16. Dwork AJ, Arango V, Underwood M, et al. Absence of histological lesions in primate models of ECT and magnetic seizure therapy. *Am J Psychiatry.* 2004;161(3):576-578.
17. Moscrip TD, Terrace HS, Sackeim HA, Lisanby SH. Randomized controlled trial of the cognitive side-effects of magnetic seizure therapy (MST) and electroconvulsive shock (ECS). *Int J Neuropsychopharmacol.* 2006;9(1):1-11.

© 2020 Weissman CR et al. *JAMA Network Open.*

18. Kosel M, Frick C, Lisanby SH, Fisch HU, Schlaepfer TE. Magnetic seizure therapy improves mood in refractory major depression. *Neuropsychopharmacology.* 2003;28(11):2045-2048.
19. Lisanby SH, Luber B, Schlaepfer TE, Sackeim HA. Safety and feasibility of magnetic seizure therapy (MST) in major depression: randomized within-subject comparison with electroconvulsive therapy. *Neuropsychopharmacology.* 2003;28(10):1852-1865.
20. White PF, Amos Q, Zhang Y, et al. Anesthetic considerations for magnetic seizure therapy: a novel therapy for severe depression. *Anesth Analg.* 2006;103(1):76-80, table of contents.
21. Sackeim HA, Prudic J, Devanand DP, et al. A prospective, randomized, double-blind comparison of bilateral and right unilateral electroconvulsive therapy at different stimulus intensities. *Arch Gen Psychiatry.* 2000;57(5):425-434.
22. Peterchev AV, Jalinous R, Lisanby SH. A transcranial magnetic stimulator inducing near-rectangular pulses with controllable pulse width (cTMS). *IEEE Trans Biomed Eng.* 2008;55(1):257-266.
23. Kirov G, Ebmeier KP, Scott AI, et al. Quick recovery of orientation after magnetic seizure therapy for major depressive disorder. *Br J Psychiatry.* 2008;193(2):152-155.
24. Oquendo MA, Malone KM, Ellis SP, Sackeim HA, Mann JJ. Inadequacy of antidepressant treatment for patients with major depression who are at risk for suicidal behavior. *Am J Psychiatry.* 1999;156(2):190-194.
25. Miller MD, Paradis CF, Houck PR, et al. Rating chronic medical illness burden in geropsychiatric practice and research: application of the Cumulative Illness Rating Scale. *Psychiatry Res.* 1992;41(3):237-248.
26. Beck AT, Kovacs M, Weissman A. Assessment of suicidal intention: the Scale for Suicide Ideation. *J Consult Clin Psychol.* 1979;47(2):343-352.
27. Chanpattana W, Sackeim HA. Electroconvulsive Therapy in Treatment-Resistant Schizophrenia: Prediction of Response and the Nature of Symptomatic Improvement. *J ECT.* 2010.
28. King MJ, Macdougall AG, Ferris SM, Levine B, Macqueen GM, McKinnon MC. A review of factors that moderate autobiographical memory performance in patients with major depressive disorder. *J Clin Exp Neuropsychol.* 2010:1-23.
29. Kennedy SH, Konarski JZ, Segal ZV, et al. Differences in brain glucose metabolism between responders to CBT and venlafaxine in a 16-week randomized controlled trial. *Am J Psychiatry.* 2007;164(5):778-788.
30. Lozano AM, Mayberg HS, Giacobbe P, Hamani C, Craddock RC, Kennedy SH. Subcallosal cingulate gyrus deep brain stimulation for treatment-resistant depression. *Biol Psychiatry.* 2008;64(6):461-467.
31. Nobler MS, Oquendo MA, Kegeles LS, et al. Decreased regional brain metabolism after ect. *Am J Psychiatry.* 2001;158(2):305-308.
32. Nordanskog P, Dahlstrand U, Larsson MR, Larsson EM, Knutsson L, Johanson A. Increase in hippocampal volume after electroconvulsive therapy in patients with depression: a volumetric magnetic resonance imaging study. *J ECT.* 2010;26(1):62-67.

© 2020 Weissman CR et al. *JAMA Network Open.*

33. Szabo K, Hirsch JG, Krause M, et al. Diffusion weighted MRI in the early phase after electroconvulsive therapy. *Neurol Res.* 2007;29(3):256-259.

© 2020 Weissman CR et al. *JAMA Network Open.*
